# The effects of a multispecies synbiotic on microbiome-related side effects of long-term proton pump inhibitor use: A pilot study

**DOI:** 10.1038/s41598-020-59550-x

**Published:** 2020-02-17

**Authors:** Angela Horvath, Bettina Leber, Nicole Feldbacher, Markus Steinwender, Irina Komarova, Florian Rainer, Andreas Blesl, Vanessa Stadlbauer

**Affiliations:** 1 Division of Gastroenterology and Hepatology, Medical University of Graz, Graz, Austria; 2 Center for Biomarker Research in Medicine (CBmed), Graz, Austria; 3 Division of Transplantation Surgery, Medical University of Graz, Graz, Austria

**Keywords:** Microbiota, Nutrition, Quality of life

## Abstract

Side effects of proton pump inhibitors (PPI) can be linked to the changes in the intestinal microbiome that occur during therapy, especially in long-term users. Therefore, the microbiome might also be a key player in the reduction of PPI side effects. We tested the effects of a three-month intervention with a multispecies synbiotic on intestinal inflammation, gut barrier function, microbiome composition, routine laboratory parameters and quality of life in patients with long-term PPI therapy. Thirty-six patients received a daily dose of a multispecies synbiotic for three months and were clinically observed without intervention for another three months. After intervention 17% of patients reached normal calprotectin levels; the overall reduction did not reach statistical significance (−18.8 ng/mg; 95%CI: −50.5; 12.9, p = 0.2). Elevated zonulin levels could be significantly reduced (−46.3 ng/mg; 95%CI: −71.4; −21.2; p < 0.001). The abundance of *Stomatobaculum* in the microbiome was reduced and *Bacillus* increased during the intervention. Furthermore, albumin, alkaline phosphatase and thrombocyte count were significantly increased and aspartate transaminase was significantly decreased during intervention. Gastrointestinal quality of life showed significant improvements. In conclusion, microbiome-related side effects of long-term PPI use can be substantially reduced by synbiotic intervention. Further studies are warranted to optimize dosage and duration of the intervention.

## Introduction

In the last 30 years proton pump inhibitors (PPI) have established themselves as highly effective treatment of acid related diseases and became the most often prescribed class of drugs in gastroenterology^1,2^. Underneath their immaculate image of gastric protection, however, their side effect profile - although well described by high quality data - remained largely unaddressed^3^. Many of the side effects might be rooted in PPI-induced changes of the microbiome (i.e. dysbiosis): increased risk of enteric infections, bloating, flatulence, abdominal pain, stool frequency abnormalities, intestinal inflammation and vitamin B12 malabsorption^4–12^.

Long term PPI use can introduce ample alterations to the intestinal microbiome: Changes in composition, reduction of alpha diversity, small intestinal bacterial overgrowth and the manifestation of oral bacteria in more distal parts of the intestine have been observed^13–19^. A loss of alpha diversity and therefore a loss in pathogen-colonization resistance could account for the increased risk of enteric infections^20,21^. The change of composition, which includes the reduction of Clostridiales and the increase of Enterococcaceae and Streptococcaceae, and function of the microbiome might directly predispose for *Clostridium difficile* infections^22^. In children, persistent bowel symptoms during PPI use have been linked to small intestinal bacterial overgrowth^23^. The association with the microbiome could be a crucial factor for the amelioration of PPI side effects. Modulating the microbiome with probiotic bacteria might be able to reduce the burden of side effects during PPI therapies. The merit of this idea has been shown in a trial administering *Lactobacillus reuteri* together with PPI that could successfully reduce small intestinal bacterial overgrowth in children^24^. Probiotics also have additional effects of which PPI-treated patients could profit: they have been shown to reduce pathogen growth and drug-induced diarrhoea, ameliorate bowel symptoms, and improve gut barrier and liver function in previous reports^25–35^. Furthermore, the use of prebiotics (i.e. indigestible dietary fibre) or synbiotics (i.e. combination of probiotics and synbiotics) can support the resident microbiome and complement the effects of probiotic supplementation^36^. However, since PPI-specific data is still scarce, the routine use of probiotics during PPI therapy is not recommended yet^1^.

Therefore, we tested the hypothesis that the administration of probiotics reduces PPI side effects. We aimed to show the effects of a three-month intervention with a multispecies synbiotic on intestinal inflammation, gut barrier function, microbiome composition, routine laboratory parameters and gastrointestinal quality of life in patients with long-term PPI therapy.

## Results

### Patients

**Figure 1:**
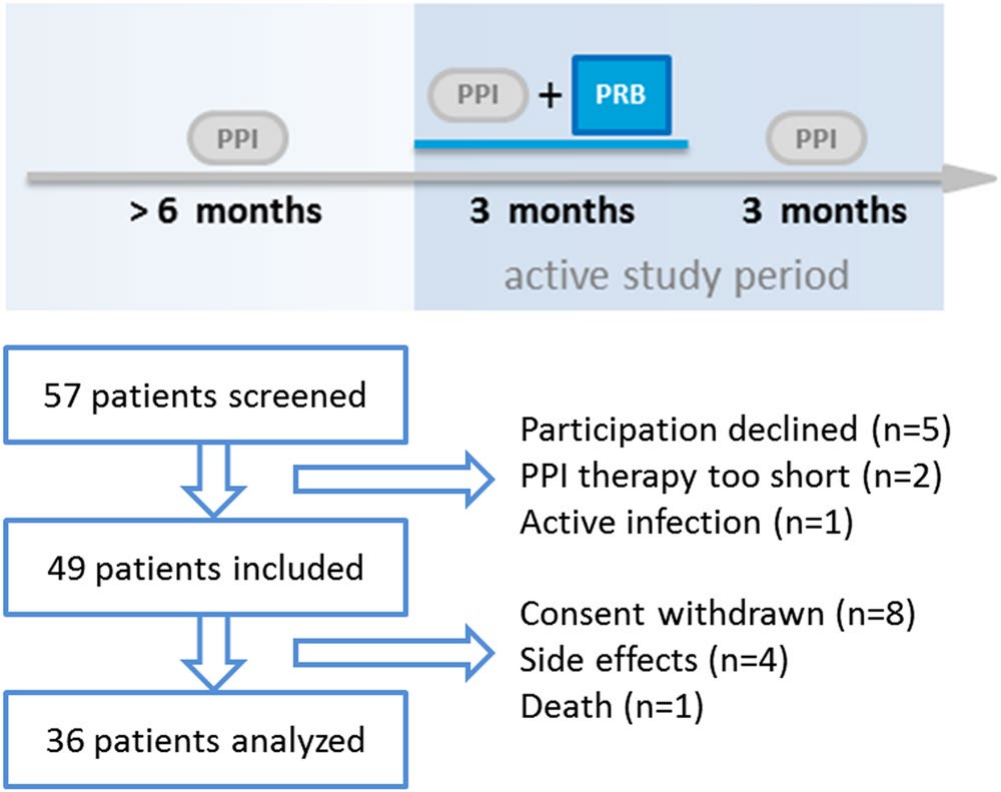
Enrolment scheme and time line.

Fifty-seven patients with long-term PPI use were screened for the study; eight patients did not meet the inclusion/exclusion criteria (declined to participate: n = 5; PPI therapy to short: n = 2; active infection: n = 1). Forty-nine patients started the intervention and 36 finished it according to the protocol. Reasons for drop-out were withdrawn consent (n = 8), side-effects (gastric pain: n = 1; gastrointestinal discomfort: n = 2; constipation: n = 1) and liver transplantation (n = 1). See also Fig. 1. Patients were on average 63 years (95%CI: 59; 67) old, 47% were female and the average duration of PPI therapy was 63 months (95%CI: 44; 82). Reasons for PPI therapy were peptic ulcer/reflux disease (n = 21), polypharmacy (n = 8) and others (n = 7). Of the 36 analyzed patients, twelve had liver cirrhosis (details are given in Table S1). Patients stayed on their PPI regime throughout the study and did not make substantial changes to their diet. Patients’ characteristics are given in Table 1.

**Table 1:** Characteristics of patients included in the analysis. (PPI: proton pump inhibitor).

	Analyzed patients (n = 36)
Age (years)	63 (59; 67)
Sex (f/m)	17/19 (47/53%)
Duration of PPI (months)	63 (44; 82)
**PPI regime:**	
Pantoprazole	24 (67%)
−20 mg	3 (8%)
−40 mg	20(56%)
−60 mg	1 (3%)
Esomeprazole	7 (19%)
−20 mg	2 (5%)
−40 mg	5 (14%)
others	5 (14%)
**Reason for PPI:**	
Peptic Ulcer/Reflux	21 (58%)
Drug use/Polypharmacy	8 (22%)
others	7 (20%)
Cirrhosis (yes/no)	12/24 (33/67%)

### Intestinal inflammation

Fecal calprotectin levels at baseline were elevated to 147.1 ng/mg (95%CI: 106.4; 187.8) and decreased on average by 18.8 ng/mg (95%CI: −50.5; 12.9, p = 0.2) during the intervention. Thirty patients (83%) had elevated calprotectin levels (i.e. >50 ng/mg) at baseline. Of these 30 patients, five (17%) showed normal calprotectin levels after the intervention, and therefore reached the primary endpoint. In total, 57% of patients with initially increased calprotectin levels showed a reduction of at least 10% after intervention. Details are given in Table 2.

**Table 2:** Baseline values and changes in biomarkers for intestinal inflammation and permeability, liver function and Vitamin B12 levels throughout the study; data are given as mean (95% confidence interval), [^a^all patients, ^b^selected patients with increased baseline values].

	Before Intervention	After Intervention	Follow up	p-value (Before vs. After)	p-value (Before Intervention vs. Follow-up)
Calprotectin (ng/mg)^a^	147.1 (106.4;187.8)	128.3 (92.1; 164.5)	142.9 (107.3; 178.4)	0.192	—
- when >50 ng/mg at baseline^b^	168.7 (123.7; 213.7)	142.6 (101.0; 184.2)	161.5 (122.1; 200.9)	0.090	—
Zonulin (ng/mg)^a^	60.9 (44.4; 77.5)	51.2 (41.8; 60.6)	51.9 (40.0; 63.8)	0.385	—
- when >50 ng/mg at baseline^b^	100.1 (73.8; 126.4)	53.8 (41.5; 66.0)	48.7 (33.7; 63.8)	<**0.001**	**0.007**
Lipopolysaccharide (EU/ml)	1.9 (0.9; 2.9)	1.6 (0.9; 2.3)	2.3 (1.2; 3.4)	0.428	—
Lipopolysaccharide-binding protein (ng/ml)	14.8 (12.3; 17.2)	14.6 (12.8; 16.4)	12.9 (10.9; 15.0)	0.561	—
sCD14 (µg/ml)	2.3 (2.1; 2.6)	2.5 (2.2; 2.8)	2.1 (1.9; 2.2)	0.177	—
Albumin (g/dl)	4.1 (4.0; 4.2)	4.2 (4.1; 4.3)	4.2 (4.1; 4.4)	**0.027**	**0.013**
Alanine aminotransferase (U/l)	29.3 (24.2; 34.3)	27.8 (23.4; 32.1)	25.5 (22.4; 28.7)	0.317	—
Aspartate transaminase (U/l)	32.0 (27.9; 36.1)	29.1 (25.2; 33.1)	28.1 (23.9; 32.3)	**0.016**	**0.005**
Alkaline Phosphatase (U/l)	76.1 (67.0; 85.1)	79.9 (70.9; 89.0)	79.6 (69.5; 89.7)	**0.004**	**0.006**
Thrombocytes (G/l)	196 (163; 229)	207 (174; 240)	216 (185; 247)	**0.010**	**0.138**
PZINR	1.1 (1.0; 1.2)	1.1 (1.0; 1.2)	1.0 (0.9; 1.1)	0.695	—
Vitamin B12 (ng/l)	406 (355; 457)	416 (362; 469)	445 (382; 508)	0.784	—
C-reactive proteins (mg/l)	3.4 (2.0; 4.9)	3.0 (2.1; 3.9)	3.8 (2.2; 5.5)	0.388	—

Since one third of the study cohort had liver cirrhosis as underlying disease, liver cirrhosis was analyzed as a potential confounder. Calprotectin levels were significantly higher in patients with cirrhosis as shown in Table S1, and the mean decrease after synbiotic intervention was larger in cirrhotic patients compared to liver healthy patients, although not statistically significant (−35.9 ng/mg (95%CI: −129.0; 57.2) vs. −10.2 ng/mg (95%CI: −32.9; 12.4), respectively, p = 0.07).

### Gut permeability

Fecal zonulin levels were slightly increased at baseline (60.9 ng/mg, 95%CI: 44.4; 77.5) with 44% of patients showing increased levels (i.e. >50 ng/mg). Patients with zonulin levels > 50 ng/mg at baseline showed significant reduction of −46.3 ng/mg (95%CI: −71.4; −21.2; p < 0.001) during intervention and maintained low zonulin levels for three months after intervention had ended. When patients with elevated and normal zonulin levels were analyzed together, the reduction did not reach statistical significance (p = 0.4). There was no change in lipopolysaccharide, lipopolysaccharide-binding protein or sCD14 levels. For details see Table 2. Zonulin levels at baseline were similar in patients with and without liver cirrhosis and both groups showed comparable decrease during synbiotic intervention (−18.8 ng/mg (95%CI: −55.8; 18.25) vs. −5.2 ng/mg (95%CI: −24.5; 14.1), respectively, p = 0.5).

### Gut microbiome

Patients showed microbiome changes consistent with the literature. Compared to a published control cohort, alpha diversity of patients before the intervention was significantly reduced (Chao1: p < 0.001). See also Fig. S1A. Beta diversity was significantly influenced by PPI use as assessed by redundancy analysis (RDA: Variance = 26.26, F = 1.7, p = 0.001) and shown in Fig. S1B. Since one third of the study cohort had liver cirrhosis as underlying disease, liver cirrhosis was analyzed as a potential confounder. Alpha diversity was not significantly different between patient groups, although liver healthy patients showed a somewhat higher Chao1 index as cirrhotic patients (p = 0.07). Beta diversity showed slight changes between the groups (RDA: Variance = 20.04, F = 1.3, p = 0.04). Details are given in Fig. S2. When underlying disease was used as a confounder in LEfSe analysis, PPI users still showed a significantly higher abundance of oral bacteria, such as *Streptococcus*, *Lactobacillus*, *Veillonella* and *Rothia* compared to controls (full list is provided in Fig. S2).

**Figure 2:**
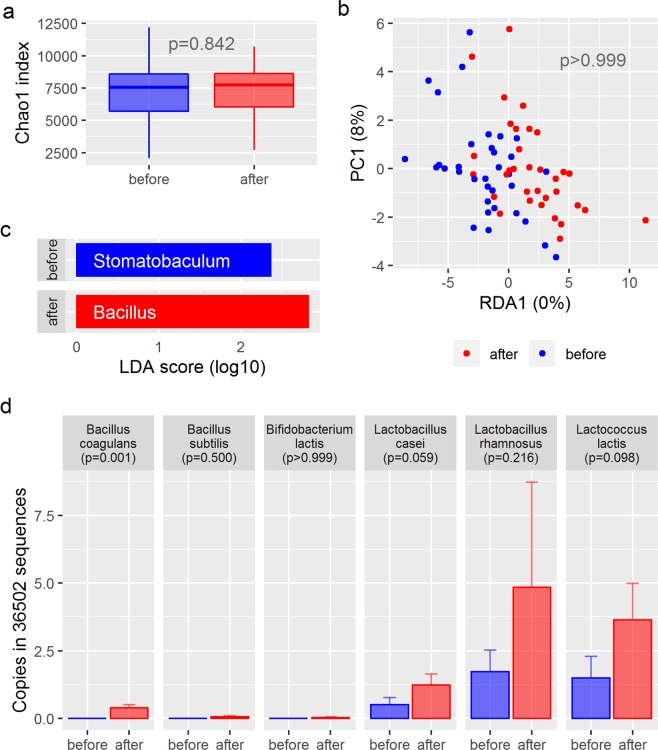
Changes in the microbiome of patients from baseline to end of intervention. (**a**) Chao1 index as a measurement for alpha diversity before and after intervention compared by paired t-test; (**b**) Redundancy analysis plot showing minimal changes in the overall beta-diversity of the microbiome during intervention. (**c**) LEfSe-Plot attributing the genus *Stomatobaculum* to pre-intervention samples and *Bacillus* to post-intervention samples. (**d**) Recovery rate of sequences matching the probiotic bacteria of the study product compared by Wilcoxon rank sum test. Bacteria not shown in this graph were not recovered.

Microbiome composition was not substantially changed after synbiotic intervention. There was no change in alpha diversity as shown in Fig. 2a (Chao1: p = 0.8). Redundancy analysis showed no specific clustering of samples before and after intervention (p > 0.999, Fig. 2b) and no distinct networks could be identified for the two time points. LEfSe attributed the genus *Stomatobaculum* to samples before intervention and the genus *Bacillus* to samples after intervention (Fig. 2c). In addition, the family Bacillaceae and an operational taxonomic unit identified as *Bacillus coagulans* were significantly increased after intervention (p < 0.001 and p = 0.001, respectively). *Bacillus coagulans* was part of the synbiotic formulation used in this study. The other probiotic strains did not show a significant increase during intervention using 16S rRNA gene sequencing technology (Fig. 2d). No other changes in the microbiome composition could be observed (see Figs. S3 and S4).

### Biochemical parameters

Albumin concentration increased significantly (+0.08 g/dl, 95%CI: 0.01; 0.15; p = 0.03) and aspartate transaminase (AST) concentration decreased significantly after treatment (−2.9U/l, 05%CI: −5.7; −0.1; p = 0.02). Alkaline phosphatase (AP) increased significantly, however within the normal range (+3.9 U/l, 95%CI: 1.2; 6.5; p = 0.004). Changes in albumin, AST and AP were sustained throughout the follow-up period. Thrombocyte count increased slightly after intervention (+11.3G/l, 95%CI: 3.3; 19.3; p = 0.01) but did not exceed the normal range in any case. Thrombocyte count at follow up was unchanged compared to baseline values. Vitamin B12 did not show any changes, however none of the patients had a vitamin B12 deficiency. None of the other biochemistry results were affected by synbiotic intervention. Details are given in Table 2.

AST and AP were significantly higher, and albumin and thrombocyte count were significantly lower in patients with cirrhosis as expected (see Table S1). Nevertheless, mean changes from baseline were comparable between patients with and without cirrhosis (Albumin: 0.05 g/dl (95%CI: −0.06; 0.2) vs. 0.1 g/dl (95%CI: 0.01; 0.2), respectively, p = 0.5; AST: −2.0U/l (95%CI: −9.8; 5.8) vs. −3.3 U/l (95%CI: −5.7; −0.9), respectively, p = 0.5; AP: 4.6 U/l (95%CI: −0.2; 9.4) vs. 3.5 U/l (95%CI: 0.06; 6.9), respectively, p = 0.6; thrombocyte count: 7.2G/l (95%CI: 0.5; 13.9) vs. 13.3G/l (95%CI: 1.6; 24.9), respectively, p = 0.4)

### Quality of life

Gastrointestinal quality of life index (GIQLI) improved significantly during the intervention (total score: +8.9 points, 95%CI: 4.0; 13.8, p = 0.001). This was mainly facilitated by an improvement in quality of life related to symptoms and emotions which showed an increase of 4.5 points (95%CI: 1.8; 7.3, 0.002) and 1.2 points (95%CI: 0.3; 2.1, p = 0.01), respectively. While quality of life related to emotions returned to baseline values three months after the intervention had ended, quality of life related to gastrointestinal symptoms remained improved until the end of the follow-up period. The short-form (SF) 36 items and the remaining items of GIQLI remained unchanged throughout the study period. For details see Table 3.

**Table 3:** Changes in gastrointestinal and health related quality of life throughout the study; data are given as mean (95% confidence interval). (GIQLI: gastrointestinal quality of life index; SF36: Short Form 36).

	Before Intervention	After Intervention	Follow up	p-value (Before vs. After)	p-value (Before Intervention vs. Follow-up)
**GIQLI**					
Emotions	14 (12; 15)	15 (14; 17)	14 (13; 16)	**0.010**	0.719
Medical treatment	3 (3; 4)	4 (3; 4)	3 (3; 3)	0.101	—
Physical function	16 (13; 18)	17 (16; 19)	16 (14; 18)	0.089	—
Social function	12 (11; 13)	13 (12; 14)	11 (10; 12)	0.060	—
Symptoms	54 (50; 57)	58 (55; 61)	55 (52 (59)	**0.002**	**0.028**
Total score	98 (92; 105)	107 (101; 113)	100 (93; 107)	**0.001**	0.159
**SF-36**					
General health	58 (51; 66)	57 (51; 64)	52 (45; 59)	0.600	—
Social role	80 (67; 92)	85 (74; 96)	76 (61; 91)	0.507	—
Physical role	65 (51; 80)	58 (43; 73)	55 (38; 72)	0.233	—
Bodily pain	60 (50; 71)	67 (58; 76)	53 (43; 64)	0.124	—
Physical functioning	75 (67; 82)	76 (69; 82)	66 (59; 74)	0.688	—
Mental health	70 (62; 77)	73 (66; 79)	69 (62; 75)	0.263	—
Social functioning	79 (71; 88)	85 (77; 92)	73 (64; 83)	0.101	—
Vitality	54 (46; 61)	57 (51; 62)	50 (43; 57)	0.492	—

Cirrhosis did not affect GIQLI scores and their average improvements during synbiotic intervention were comparable between patients with and without cirrhosis (total score: 9.6 points (95%CI: −0.6; 19.7) vs. 8.6 points (95%CI: 2.6; 14.6), respectively, p = 0.6; symptoms: 4.8 points (95%CI: −1.5; 9.9)vs. 4.7 points (95%CI: 1.3; 8.1), respectively, p = 0.9; emotions: 0.5 points (95%CI: −1.1; 2.0) vs. 1.6 points (95%CI: 0.47; 2.7), respectively, p = 0.4).

## Discussion

This study shows that a multispecies synbiotic containing twelve strains has the potential to improve PPI side effects after three months of intervention. We observed improvements in gut permeability, liver biochemistry parameters and quality of life; however intestinal inflammation and microbiome composition were unchanged.

Calprotectin levels were increased in the study patients, who were all free of any inflammatory bowel disease. This confirms previous reports that linked PPI use and PPI-associated changes in the microbiome to increased calprotectin levels, irrespective of underlying conditions^11,12,37^. Although some improvement in intestinal inflammation was observed, the primary endpoint that was defined as a normalization of elevated calprotectin levels was not reached. We were able to achieve a 10% reduction of calprotectin levels in 57% of the patients. It is yet unclear whether calprotectin needs to normalize in order to prevent PPI induced side effects or if a reduction will also provide clinical benefits. Since the average duration of PPI therapy was 63 months, a three-month synbiotic intervention during ongoing PPI treatment might also be too short to ameliorate inflammatory conditions.

On the other hand, zonulin levels were responsive to the intervention and, when elevated at baseline, showed significant reduction after three months and did not increase three months after the synbiotic intervention had stopped. The regulatory mechanism of probiotics on zonulin levels have been shown in trained men after a 14 week long intervention with a multispecies probiotic. Simultaneously, a decrease in TNFα and a reduction of exercise-induced protein oxidation was observed^30^. Also, perioperative interventions with multispecies probiotics in patients with colorectal cancer and/or colorectal liver metastases could reduce zonulin levels and reduce post-operative bacterial translocation, infectious events and duration of antibiotic therapy^38,39^. A probiotic intervention in cirrhosis failed to reduce zonulin levels after three and six months^40^, however, patients’ zonulin levels in that study were comparable to healthy controls. A recent report linked zonulin levels in cirrhosis to PPI-associated dysbiosis^37^.

Dysbiosis is well described in PPI users; the main features are a loss in diversity, oralization and small intestinal bacterial overgrowth^13–15,18^. Patients in our study exhibited typical changes in the composition of their microbiome compared to healthy controls without PPI use (details are given as Supplementary Information). Foremost, the increase in predominantly oral bacteria (i.e. oralization), including Streptococcus, Veillonella, Rothia, among others, is evident, as it has been described previously for PPI users^15,37^. Furthermore, the decrease of beneficial genera has been observed in our patients in accordance to the literature^17,19^. Unfortunately, changes in the microbiome composition due to the intervention were minimal after three months. Two genera were differentially abundant between the time points, *Stomatobaculum* and *Bacillus*. *Stomatobaculum* is an anaerobic bacterium from dental plaques^41^. The occurrence of this bacterium in stool can be a sign of oralization of the intestinal microbiome during PPI use. The abundance of *Stomatobaculum* was reduced during the intervention which might hint towards a potential role of probiotics in correcting PPI-induced dysbiosis (i.e. oralization), although *Stomatobaculum* is not a very prevalent oral taxon found in the intestinal microbiome of PPI users.

*Bacillus* increased during intervention, probably due to *Bacillus coagulans* that was ingested with the study product. Out of the twelve probiotic strains, only *Bacillus coagulans* was significantly increased. We were previously able to recover more strains in stool of patients with liver cirrhosis who used Ecologic Barrier/OmniBiotic Hetox (Winclove, Amsterdam, The Netherlands/Institut Allergosan Graz, Austria) - a product that shares 7 strains with the product under investigation in this study^42^. The reason for this difference is still elusive. The formulation and concentration of the study products differ between trials as well as the duration of intervention and the patient collective. Probiotic formulations have a wide range of bacterial content and evidence for dose dependent effects is still scarce^43^. Different formulations have different minimum concentrations to be effective; in irritable bowel syndrome, *B. infantis* 35624 could reduce symptoms at a daily dose of 10^8 CFU while higher dosages did not bring any additional benefit^44^. Other formulations on the other hand, do show dose dependent increases in efficacy^45^. The daily dose of bacteria in the study product with a total bacterial load of 8 × 10^9^ was lower than other popular probiotics such as the original VSL#3 (Alphasigma, Covington, USA) or Ecologic Barrier (Winclove, Amsterdam, The Netherlands). These formulations contain 5 × 10^11^ and 1.5 × 10^10^ CFU per dose respectively and consist of eight/nine distinctive probiotic strains compared to twelve strains in the study product^40,46^. It is therefore possible that the concentration of each individual strain was too low to be detected by the sequencing effort in this study. Whether a higher dose of the study product could intensify the effects in patients with long-term PPI therapy, can only be clarified in additional clinical studies.

Furthermore, we observed a slight but significant increase in albumin levels and a decrease in the levels of the liver enzyme AST. The positive effects of probiotics on AST in non-alcoholic fatty liver disease are well documented^47,48^. Moreover, probiotic interventions in patients with and without different liver diseases were also attested a significant reduction of AST^49^. Albumin is an indicator for liver function and for the absence of inflammation. Besides liver function, albumin also reflects the nutritional status of a patient. However, nutritional habits stayed unchanged throughout the study and the patients were all well-nourished, excluding nutrition as a possible reason for the increase in albumin in this study. Alkaline phosphatase also increased significantly during the intervention and higher levels were sustained during the follow-up period. Only 3/36 measurements were outside the normal range at the end of intervention, all 3 were within 1.5x of the upper limit of normal and gamma-glutamyl-transferase did not increase. This excludes cholestatic liver damage as possible source for the increase in AP. Human AP in serum is a mixture of several isoenzymes with different functions and AP levels can also be influenced by age, diet and various diseases^50^. Serum AP outside the normal range has been associated with higher mortality^51,52^. However, other studies show that AP can also exert beneficial effects. In animal models of necrotizing enterocolitis, intestinal AP administration could reduce systemic inflammation^53^ and protect gut barrier function in neonatal rats^54^. Moreover, *in-vitro* studies have indicated intestinal AP in the up-regulation of tight junction proteins^55^. Recombinant AP is under investigation to prevent sepsis-associated acute kidney injury in humans^56^, however, the phase II study failed to show a clinical benefit^57^. In addition to mammalian isoenzymes, several probiotic strains in the study product have shown substantial AP activity, which might contribute to the detoxification of lipopolysaccharide^58^. Whether these enzymes translocate through the intestinal barrier and contribute to the total serum AP activity is not clear. A more comprehensive study of AP isoenzymes is warranted to answer this question. Taken together the changes in AST and albumin speak to the hepatoprotective effect of probiotics that has been indicated in previous reports^40,59,60^.

Quality of life is an important outcome assessor and reflects the impact of health care interventions on the patients’ everyday life and well-being^61,62^. The improvement in quality of life in the present study was based on a reduction of bowel symptoms and negative emotions. This reflects previously shown positive effects of probiotics on bowel symptoms in irritable bowel syndrome and functional bowel disorder^63–65^. *Bacillus coagulans* as a single-strain synbiotic formulation has been shown to reduce abdominal pain and diarrhea in irritable bowel syndrome patients^66^. *Bacillus coagulans* was also the most prominent probiotic strain in the presented study, which sparks interest in the modes of action of this probiotic bacterium. The microbiome can influence quality of life and features of depression, and the potential to improve sad mood by modulating the microbiome with probiotics has been demonstrated before^67,68^. This is in accordance to the improved quality of life in regards to emotions that has been observed in the presented study. There were no changes in quality of life measured with the SF-36, although both questionnaires deal with patients’ emotions. A previous report by Shi *et al*. found that even though both instruments show validity on their own and there are clear correlation between the outcomes of the two instruments, GIQLI showed superior responsiveness in regards to emotional well-being in patients with cholecystectomy^69^. It, therefore, seems to be more suitable for studying the impact of interventions in gastrointestinal diseases. Regardless of the questionnaire, quality of life is a subjective measure and the effects observed in this study need to be validated in a blinded, controlled trial.

To our knowledge this is the first study analyzing the effect of a synbiotic intervention on intestinal inflammation, gut permeability, microbiome composition, liver function and quality of life in long term PPI users. So far only one small study addressed the effect of probiotics in children receiving a 12 week treatment with PPI for gastroesophageal reflux. In this study no microbiome sequencing data were presented, but small intestinal bacterial overgrowth (measured with a hydrogen breath test) could be reduced significantly^24^.

The foremost limitation of this study is the design as open labelled, uncontrolled trial. To our knowledge comparable trials in adults to address PPI-induced dysbiosis are missing. Therefore, a pilot trial was necessary on which decisions about endpoint and sample size for a full-sized randomized control trial can be based. A full-sized randomized control trial without pilot data would not have been economically feasible. This trial was supposed to detect potential targets for synbiotic interventions in patients with long-term PPI use, in order to inform upcoming trials by us and others. The primary and many of the secondary endpoints are hard endpoints likely not influenced by the lack of a placebo control; however, the chosen trial design limits the interpretation of the more subjective measures, such as quality of life. The dropout rate of 27% was higher than expected (20%). Adverse effects related to abdominal discomfort accounted for one third of drop-outs. Since we performed the study as an open-label pilot trial, we are not able to discuss the relation of the product to the side effects. In a previous study using a multispecies probiotic containing 9 strains (7 of which are also present in the study product of this study) a much lower dropout rate (2%) and no side effects were observed in the treated group; in the placebo group the drop-out rate was 23%^40^. Generally, there is little information about the side-effect profile of individual strains, therefore a meaningful discussion about adverse effects of specific probiotics in this and other trials is not yet possible and comprehensive studies are warranted.

In conclusion: Synbiotic interventions have the potential to improve side effects of long-term PPI therapy in cirrhotic and liver healthy patients. Future studies are necessary to validate the here presented benefits and may find valuable therapeutic targets in gut permeability, liver function parameters and quality of life.

## Methods

Between September 2017 and March 2018 an open-label pilot trial was conducted at the University Hospital Graz/Austria (Division of Gastroenterology and Hepatology). Patients from the department’s outpatient clinic were included in the study if they were at least 18 years old, gave written informed consent und had a history of continuous PPI use for at least 6 months. Exclusion criteria were active infections at time of inclusion, antibiotic therapy including prophylactic therapy within 14 days of inclusion, inflammatory bowel disease, consumption of pre-, pro- or synbiotics other than the study product, and concomitant diseases or circumstances that suggests that the patient is not eligible. A daily dose of 4 g of a multispecies probiotic (Institute Allergosan, Graz, Austria/Winclove Probiotics, Amsterdam, The Netherlands) was administered to all patients for three months followed by a three-month observation period without intervention. The study product contained 12 strains (*Bacillus coagulans* W183, *Bacillus subtilis* W201, *Bifidobacterium bifidum* W23, *Bifidobacterium lactis* W52, *Bifidobacterium lactis* W51, *Lactobacillus acidophilus* W37, *Lactobacillus acidophilus* W22, *Lactobacillus casei* W56, *Lactobacillus rhamnosus* W71, *Lactobacillus salivarius* W24, *Lactococcus lactis* W19, *Propionibacterium freudenreichii* W200) in a total concentration of 2 × 10^9^ cfu/g. The probiotic strains were selected based on their capacity to inhibit pathogens (*Salmonella, E. coli and C. difficile*), improve intestinal barrier function and the ability to produce vitamins. The bacteria were embedded in a prebiotic matrix of corn starch, maltodextrin, fructo-oligosaccharide P6, inulin P2 and vegetable protein. Patients dissolved the powder in 125 ml of water and drank the suspension after a 10 minute activation period.

Primary endpoint was the normalization of calprotectin levels in stool; this was also the basis for the sample size calculation. Secondary endpoints were gut permeability (assessed by faecal zonulin levels, serum levels lipopolysaccharide, lipopolysaccharide-binding protein and sCD14), microbiome composition, gastrointestinal and health-related quality of life [assessed by gastrointestinal quality of life index (GIQLI) and Short Form (SF)-36 questionnaires], vitamin B12 levels and routine laboratory parameters. Dietary habits were tracked by an extensive, validated food frequency questionnaire^70^.

The study protocol was approved by the Research Ethics Board of the Medical University of Graz (29-552 ex 16/17) and was registered prior to the inclusion of the first patient at clinicaltrials.gov (NCT03220802, 18.07.2017). All study procedures were performed according to the Declaration of Helsinki and Good Clinical Practice. For more detailed information, the study protocol is provided as Supplementary Information.

### Laboratory assessments

Commercially available ELISA were used to assess calprotectin and zonulin in stool (Immundiagnostik, Bensheim, Germany), as well as LBP and sCD14 in plasma (Hycult Biotechnology, Uden, Netherlands and R&D Systems, Abbington, UK, respectively). Lipopolysaccharide was measured with a cell-based detection system (Invitrogen, Toulouse, France) using an adapted protocol^40^. Reference values for normal calprotectin levels (<50 ng/mg) were provided by the manufacturer. Reference values for low zonulin levels (<50 ng/mg) were established in our laboratory as recommended by the manufacturer with samples from healthy volunteers with normal calprotectin levels.

### Microbiome analysis

Stool samples were collected by the patients in sterile collection tubes either on the same day or the evening before the study visit. Samples were kept on 4 °C until arrival at the hospital and then frozen immediately at −80 °C. DNA was isolated with the MagNA Pure LC DNA Isolation Kit III (Bacteria, Fungi) (Roche, Mannheim, Germany) according to manufacturer’s instructions. Hypervariable region V1-V2 was amplified (primers: 27F-AGAGTTTGATCCTGGCTCAG; R357-CTGCTGCCTYCCGTA) and sequenced using Illumina Miseq technology (Illumina, Eindhoven, Netherlands) as described before^71^. Paired end reads were then joined by fastq-join tool. Primers were removed by cutadapt 1.6 and USEARCH 6.1^72^ was used for reference based chimera detection. Open reference operational taxonomic unit picking was done with SILVA v132 as reference database. When necessary, sequences were blasted in the NCBI database for further classification^73^. Clustering was performed by UCLUST^74^ with a 97% sequence similarity threshold. Fasttree was used to generate a phylogenetic tree. After pre-processing, an average of 68 014 (range from 36 502 to 89 457) reads per sample was available. Data was normalized by rarefaction (sampling depth: 36 502 reads). Rarefied data was used for alpha diversity assessment with Chao1 index and beta diversity assessment with redundancy analysis with one or two explanatory variables. LDA effect size (LEfSe) was used to find differentially abundant taxa on family, genus and OTU level. An LDA score (log10) of 2 or higher was regarded as significant. Network analysis was done based on Spearman correlation where only positive correlations were taken into account. Data analysis was performed with QIIME1.9.1^75^ implemented on a local Galaxy instance (https://galaxy.medunigraz.at/), redundancy analysis, network analysis and linear discriminant analysis effect size (LEfSe) were done in the web-based software Calypso 8.84 (http://cgenome.net/wiki/index.php/Calypso)^76^.

In order to establish that patients included in the study show PPI-associated dysbiosis, an already published cohort^71^ of 19 healthy controls without PPI intake was used to compare the composition of the microbiome between the groups. A brief description of the control cohort is given in Table S2.

### Statistical analysis

All patients who finished the study per protocol were included in the analysis. Count data is presented as absolute numbers and percentages, continuous data as means and 95% confidence intervals. Differences between values before and after intervention/follow up were evaluated with Wilcoxon signed-rank tests. SPSS 23 (SPSS Inc., Chicago, IL, USA) was used for statistical analysis and the R packages “ggplot2” and “ggpubr” for visualization^77–79^.

### Data availability

Sequencing data is made publicly available at the NCBI Sequence Read Archive under the accession number PRJNA532853. All other data is shared upon reasonable request sent to the corresponding author.

## Supplementary information

Supplementary Information.
