# Supplementary material for: The effects of a multispecies synbiotic on microbiome-related side effects of long-term proton pump inhibitor use: A pilot study
Source: Sci Rep. 2020 Feb 17;10:2723. doi: 10.1038/s41598-020-59550-x (PMC7026433; doi:10.1038/s41598-020-59550-x)
Supplement: Supplementary file 1 — Supplementary Information. [file 41598_2020_59550_MOESM1_ESM.pdf]

Supplementary Information

The effects of a multispecies synbiotic on microbiome-related side effects of long-term proton pump inhibitor use: A pilot study

Angela Horvath,[1,2] Bettina Leber,[3] Nicole Feldbacher,[1,2] Markus Steinwender,[1] Irina Komarova,[1] Florian Rainer,[1] Andreas Blesl,[1] Vanessa Stadlbauer[1]

[1]Division of Gastroenterology and Hepatology, Medical University of Graz, Graz, Austria

[2]Center for Biomarker Research in Medicine (CBmed), Graz, Austria

[3]Division of Transplantation Surgery, Medical University of Graz, Graz, Austria

Supplementary tables:

Table S1: Patients' characteristics and baseline values of endpoints of interest according to underlying disease. Data is given as count (%) or mean (95%CI).

	Cirrhosis	Non-cirrhosis	p-value
N	12	24	
Age	58 (49; 66)	65 (61; 70)	0.112
Sex (f/m)	3/9	14/10	0.083
PPI duration (months)	43 (16; 69)	73 (47; 99)	0.092
PPI regime:			
Pantoprazole	10 (83%)	14 (58%)	0.134
-20mg	1 (8%)	5 (21%)	
-40mg	9 (75%)	8 (33%)	
-60mg		1 (4%)	
Esomeprazole	2 (17%)	5 (21%)	0.766
-20mg		2 (8%)	
-40mg	2 (17%)	3 (13%)	
others		5 (21%)	0.088
Reasons for PPI			
Peptic Ulcer/Reflux	3 (25%)	18 (75%)	**0.004**
Drug use/Polypharmacy	7 (58%)	1 (4%)	**<0.001**
others	2 (17%)	5 (21%)	0.766
MELD	11 (9; 13)	na	
Calprotectin (ng/mg)	250 (159.5; 340.6)	95.7 (69; 122.4)	**<0.001**
Zonulin (ng/mg)	61.6 (25.2; 98)	60.6 (41.4; 79.7)	0.497
Albumin (g/dl)	3.9 (3.6; 4.2)	4.2 (4.2; 4.3)	**0.005**
AST (U/l)	42.5 (34.5; 50.5)	26.8 (23.5; 30)	**<0.001**
AP (U/l)	94.5 (76.6; 112.4)	66.9 (58; 75.7)	**0.006**
Thrombocyte count (G/l)	94.8 (68.5; 121.2)	244.3 (213.8; 274.8)	**<0.001**
GIQLI – total score	91.5 (80.2; 102.7)	100.1 (92.4; 107.8)	0.198
GIQLI – symptoms	55.3 (48.4; 62.2)	52 (47.8; 56.2)	0.430
GIQLI – emotions	12.1 (9.3; 14.8)	14.4 (12.6; 16.3)	0.092

Table S2: Description of published[1] cohort of healthy controls. Data is given as mean (95%CI) or count (%)

	**healthy (n=19)**
Age (in years)	59 (56, 62)
Sex (f/m)	10/9 (53/47%)
Antibiotic use	0 (0%)
PPI use	0 (0%)

Supplementary figures

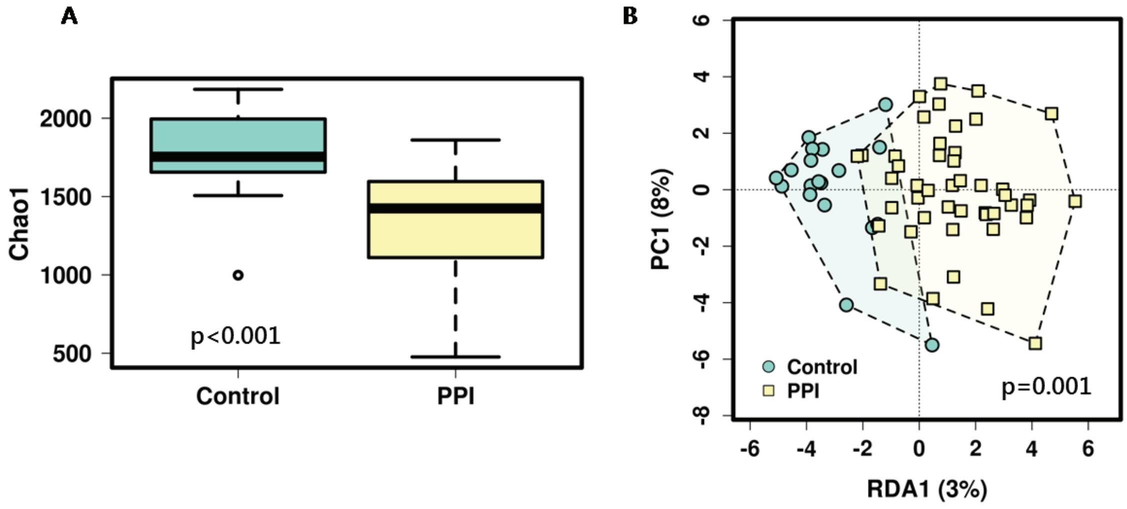

Figure S1: Alpha and beta diversity of the microbiome at baseline compared to healthy controls (n=19). A. Chao 1 index of healthy controls and PPI users; B. Redundancy analysis on the influence of PPI use on the composition of the microbiome;

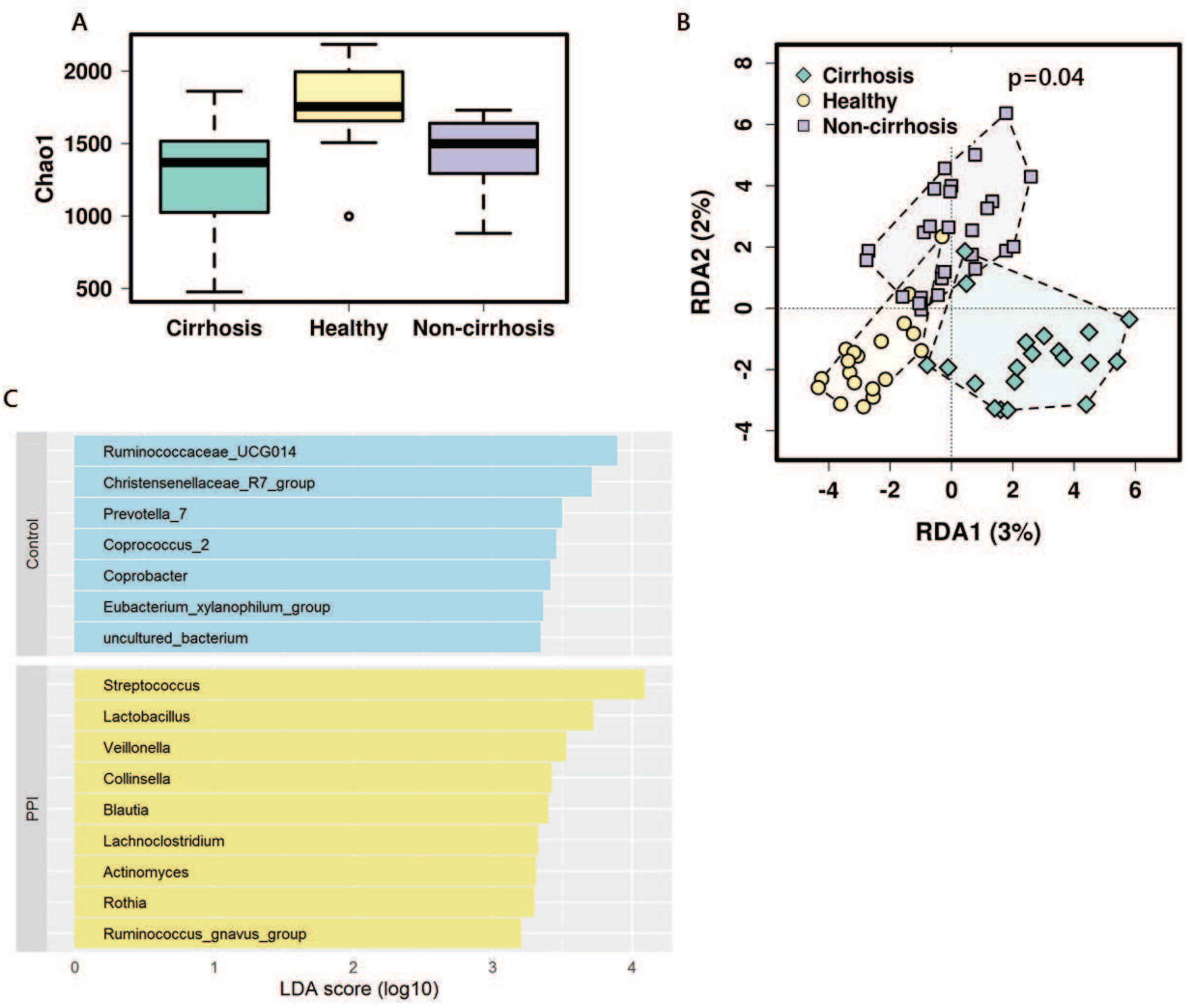

Figure 2B: The influence of underlying liver disease on microbiome composition during PPI therapy. A. Chao1 index of cirrhotic and liver healthy patients in comparison to healthy controls; B. Redundancy analysis on the influence of liver cirrhosis on the composition of the microbiome with PPI as confounder; C. LEfSe analysis to find differentially abundant genera for PPI users and controls with underlying liver disease as confounder.

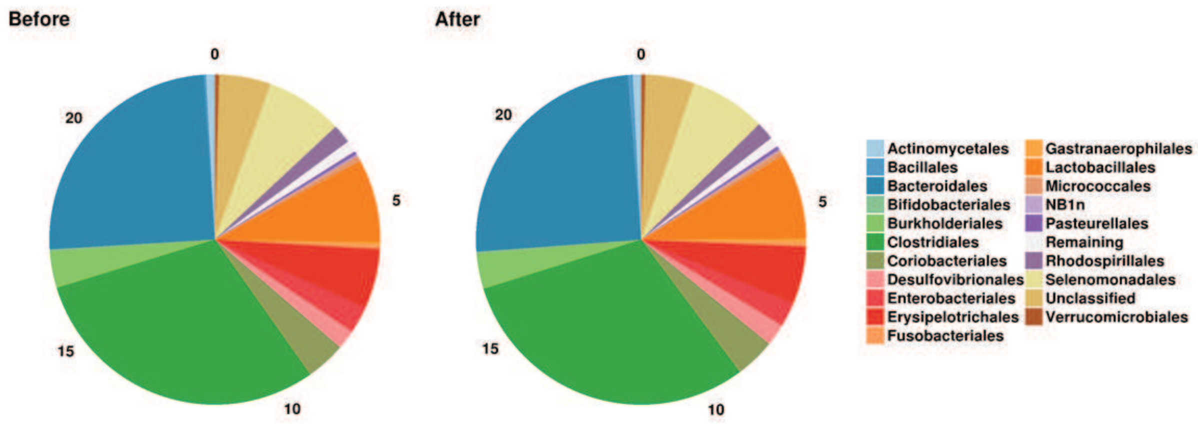

Figure S3: Composition of the microbiome on order level before and after synbiotic intervention

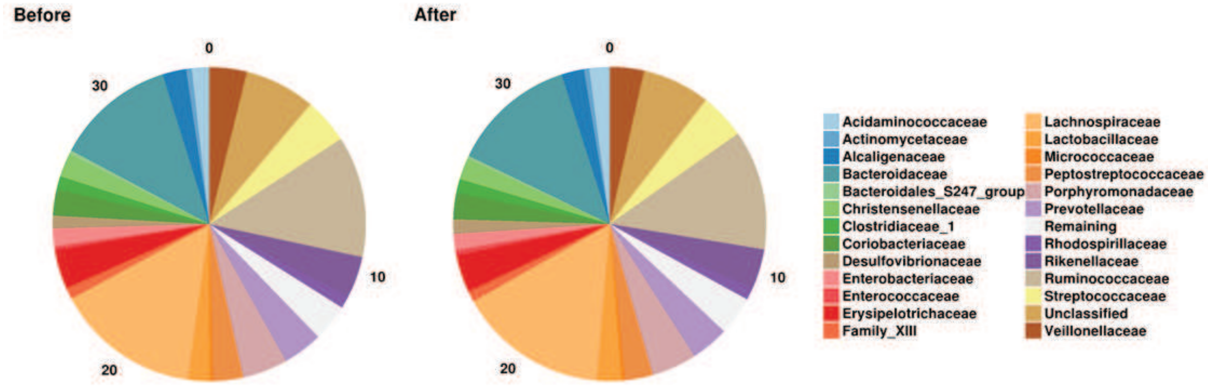

Figure S4: Composition of the microbiome on family level before and after synbiotic intervention

References

1 Stadlbauer, V. *et al.* Structural and functional differences in gut microbiome composition in patients undergoing haemodialysis or peritoneal dialysis. *Scientific reports* **7**, 15601, doi:10.1038/s41598-017-15650-9 (2017).

Probiotic modulation of intestinal microbiota in long-term intake of proton pump inhibitors: Pilot study on the influence on intestinal inflammation and digestion.

Version 3.1

15.11.2019

Principal Investigator

Assoz. Prof. Dr. Vanessa Stadlbauer-Köllner

Co-Investigators

Mag. Angela Horvath, PhD

Bianca Schmerböck, BSc

Mag. Dr. Bettina Leber

Irina Komarova, MD

Dr. Franziska Durchschein

Dr. Florian Rainer

Dr. Andreas Blesl

MSc. Andrea Streit

DGKS Andreas Posch

Table of Contents

1. General conditions ... 3
1.1. Scientific background of the study ... 3
1.2. Hypothesis and aims ... 4
1.2.1. Primary hypothesis: ... 4
1.2.2. Secondary hypotheses: ... 4
1.3. Expected effects on the advancement of clinical practice and/or patient treatment ... 5
2. Methods and research design ... 6
2.1. Type of study ... 6
2.2. Recruitment ... 6
2.2.1. Inclusion criteria ... 6
2.3. Exclusion criteria ... 6
2.4. Study product ... 6
2.5. Primary and secondary endpoints of the study ... 7
2.5.1. Primary Endpoint ... 7
2.5.2. Secondary Endpoint ... 7
2.5.3. Source Data ... 7
3. Visit Schedule ... 8
3.1. Biometric data / statistical analyses, sample size ... 10
3.1.1. Sample size ... 10
3.2. Statistical Analysis Plan ... 10
3.3. Gender aspects in research approach ... 11
3.4. Description of methods to be applied ... 11
3.4.1. Sample *acquisition* ... 11
3.4.2. Questionnaires ... 12
3.4.3. Blood sampling ... 12
3.4.4. Calprotectin: ... 12
3.4.5. Microbiome analysis: ... 12
3.4.6. LBP ... 14
3.4.7. LPS ... 14
3.4.8. Zonulin ... 15
3.4.9. sCD14 ... 15
4. Work and Time Plan ... 15
5. General rules and provisions ... 16
5.1. Ethical aspects ... 16
5.2. Legal regulations and provisions relevant to the study ... 16
6. Potential additional benefits of the study ... 16

1. General conditions

1.1. Scientific background of the study

Proton pump inhibitors (PPIs) are among the top 5 most widely used drugs in the world [1]. PPIs suppress the formation of gastric acid through the inhibition of hydrogen-potassium-adenosine-triphosphatase (H^+/ K^+-ATPase) - a known proton pump in the parietal cells of the stomach. In practice, PPIs are commonly prescribed to treat GI disorders such as peptic ulcers and gastro-oesophageal reflux [2]–[4]. They are also used prophylactically to prevent stress ulcers and to reduce GI toxicity associated with certain medications, including non-steroidal anti-inflammatory drugs (NSAIDs), aspirin, and steroids, sometimes despite a paucity of evidence [5]–[7]. PPI use has been associated with increased risk of enteric infections [8]–[10]. A meta-analysis of 23 studies, comprising almost 300.000 patients, showed a 65% increase in the incidence of *Clostridium difficile*-associated diarrhoea among patients who used PPIs [10]. Another meta-analysis of 11.280 patients, from six studies evaluating *Salmonella*, *Campylobacter* and other enteric infections, also found an increased risk due to acid suppression, with a greater association with PPI than with H2-receptor antagonists [9]. Moreover, long term PPI use has been shown to be associated with bowel symptoms: A study from 2011 reported incidences of bloating, flatulence, abdominal pain and diarrhoea (43%, 17%, 7% and 2% of selected cases, respectively) [11]. In addition, PPIs are known to cause malabsorption of Vitamin B12 [12], [13] which may ultimately lead to Vitamin B12 deficiency [14]. Very recently, PPI use was associated with an increase in mortality [15].

The gut microbiome plays an important role in enteric infections and bowel symptoms [16]–[22]. The composition of the gut microbiome can inhibit or promote the microbial colonisation of the gut by microbial pathogens. Several mechanisms can influence bacterial growth or the immune system [16], [17].

Long term PPI use is associated with profound changes in the gut microbiome [23], [24], especially in high risk populations such as patients with liver cirrhosis. It is believed that these conditions are caused through the long-term suppression of gastric acid secretion (and thus shifting intragastric pH) which alters the natural

habitat of resident microbiota. Furthermore, increased gastric pH might not sufficiently protect against oral or food-borne pathogens. We recently conducted a study at the Medical University of Graz where we could show that patients with long-term PPI therapy have dysbiosis associated with intestinal inflammation, increased gut permeability, bacterial translocation and systemic inflammation associated with a higher risk of complications and mortality in liver cirrhosis. (A. Horvath et al., Long-term proton pump inhibitor use increases intestinal dysbiosis, gut permeability, inflammation and mortality in patients with liver cirrhosis, UEG Journal, accepted)

Probiotics are live microorganisms that have been demonstrated to alter gut flora and exhibit positive effects on numerous gastrointestinal complaints, strengthen the gut barrier and reduce inflammation parameters [25]–[29] .

1.2. Hypothesis and aims

We hypothesize that a three months probiotic intervention with OMNi BiOTiC PPI improves PPI induced dysbiosis, intestinal inflammation and gut permeability in patients on long term PPI therapy, leading to a decrease in bacterial translocation and a better gastrointestinal quality of life.

1.2.1. Primary hypothesis:

A three months probiotic intervention with OMNi BiOTiC PPI reduces elevated faecal calprotectin levels in patients with long-term PPI use.

1.2.2. Secondary hypotheses:

A three months probiotic intervention with OMNi BiOTiC PPI improves PPI associated *Vellonella/Streptococcus* dysbiosis index in patients with long-term PPI use.

A three months probiotic intervention with OMNi BiOTiC PPI reduces gut permeability and bacterial translocation in patients with long-term PPI use.

Circulating vitamin B12 levels are improved by a three months probiotic intervention with OMNi-BiOTiC PPI in patients with long-term PPI use.

1.3. Expected effects on the advancement of clinical practice and/or patient treatment

From this study, we hope to learn if PPI associated side effects can be reverted through the intake of a probiotic product. This will help to develop future treatments to minimize gastro-intestinal side effects caused by PPIs.

2. Methods and research design

2.1. Type of study

An open pilot study will be conducted. The study protocol will be registered at ClinicalTrials.gov.

2.2. Recruitment

Fifty patients will be recruited from outpatient clinics at the Department of Gastroenterology and Hepatology at the Medical University of Graz. Each patient will be followed for 6 months.

2.2.1. Inclusion criteria

Age >18

Consent

PPI intake for at least 6 months

2.3. Exclusion criteria

Active infections at time of inclusion

Antibiotic therapy within the last 14 days (includes prophylactic use)

Inflammatory bowel diseases

Consumption of pre/synbiotics other than the product provided during the trial

Concomitant diseases or other circumstances that suggest that the patients are not eligible for participation in the study

2.4. Study product

The study product consist of a sachet containing 4g of yellowish powder made of corn starch, maltodextrin, fructo-oligosaccharide P6, inulin P2, vegetable protein and 12 bacterial strains (*Bacillus coagulans* W183, *Bacillus subtilus* W201, *Bifidobacterium bifidum* W23, *Bifidobacterium lactis* W52, *Bifidobacterium lactis* W51, *Lactobacillus acidophilus* W37, *Lactobacillus acidophilus* W22, *Lactobacillus casei* W56, *Lactobacillus salivarius* W24, *Lactococcus lactis* W19, *Propionibacterium freudenreichii* W200, *Lactobacillus rhamnosus* W71, in a concentration of 2×10^9 cfu/g). The study product contains 20 kilocalories per sachet. The powder has to be

dissolved in 125 ml of water for 10 minutes and should be consumed immediately after stirring.

2.5. Primary and secondary endpoints of the study

2.5.1. Primary Endpoint

Changes in faecal calprotectin levels over three months due to the probiotic intervention.

2.5.2. Secondary Endpoint

The influence of the probiotic intervention on the composition of faecal microbiome, PPI-associated *Veillonella/Streptococcus* dysbiosis index, faecal zonulin levels (biomarker for intestinal hyperpermeability), serum concentrations of LBP, sCD14 and LPS (biomarker for bacterial translocation), gastrointestinal complaints, quality of life and circulating vitamin B12 levels.

2.5.3. Source Data

Source documents comprise the CRF and hospital records, laboratory records and correspondence. All documents will be stored safely in a confidential manner. Patients will be given a study code when included in the study. This code is used to identify study-relevant documents. A list, which identifies the patients on the basis of the study codes, is led by the investigator. Source data will be made available for internal and external audits or inspections by regulatory authorities to authorised personnel only.

3. Visit Schedule

Table 1: Schedule of assessment

**Visit no.**	**1**	**2**	**3**
**Timing**		3 months +/- 7 days	6 months +/- 7 days
Inclusion / exclusion criteria	X		
Demographics/relevant medical history/current medical conditions	X	X	X
Standard laboratory tests	X	X	X
Blood sampling for gut permeability, bacterial translocation, Vitamin B12	X	X	X
Stool sampling for microbiome analysis, intestinal inflammation and gut permeability	X	X	X
Questionnaires	X	X	X
Dosage administration, adverse events, Concomitant medications/therapies	Ongoing data capture		
Study Completion	Complete at any time if study drug is discontinued		

Visit 1 Screening and baseline

Patients with long-term PPI intake (>6 months) will be eligible for the study. Those who accomplish the inclusion criteria and none of the exclusion criteria will be enrolled in the trial and submitted to the following investigation procedures in the screening visit, after signing the informed consent:

- Check inclusion and exclusion criteria
- Check for demographic data, medical and surgical history and concomitant medication
- Date of birth, gender, smoking status, alcohol consumption
- Body weight and body height
- Vital signs: resting pulse and blood pressure
- Blood sampling
- Stool sampling
- GI symptoms, quality of life
- Distribution of the study product

Visit 2 Follow up 1 (3 months +/-7 days after V1)

As part of this visit the following procedures will be performed:

- Medical history, changes in medication, adverse events
- Body weight
- Vital signs: resting pulse and blood pressure
- Blood sampling
- Stool sampling
- GI symptoms, quality of life

Visit 3 Follow up 2 (6 months +/- 7 days after V1)

As part of this visit the following procedures will be performed:

- Medical history, changes in medication, adverse events
- Body weight
- Vital signs: resting pulse and blood pressure

- Blood sampling
- Stool sampling
- GI symptoms, quality of life

3.1. Biometric data / statistical analyses, sample size

3.1.1. Sample size

The majority (89%) of patients who take PPIs and who exhibit PPI-associated *Veillonella/Streptococcus* dysbiosis have values of over 88 ng/mg stool (Horvath et al, submitted). In patients without PPI induced dysbiosis only 24% have calprotectin levels above 88 ng/ml. Using McNemar's Z-test with an alpha of 5% and a beta of 20%, we need 19 patients for this study to show this difference. We expect a dropout rate of 20%. Since the main indications for PPI use are either reflux disease or liver cirrhosis and the microbiome in these two groups is expected to be significantly different, we would like to study these two groups separately, we will include 50 patients in total. The calculation was done using www.powerandsamplesize.com.

3.2. Statistical Analysis Plan

The study consists of three time points and one study group. Therefore, the majority of tests will be comparisons of repeated measurements. After the active part of the study, the first two time points will be compared to evaluate the effect of the probiotic intervention. At the end of the study, including observation period, a complete analysis will be conducted.

Knowing from previous microbiome studies that normal distribution in these measurements is rare, non-parametric tests are described here. However, Kolmogorov-Smirnov test will be conducted to test for normal distribution. If measurements are normally distributed, parametric tests will be used.

For all non-microbiome data:

Comparison of paired categorical variables will be done using McNemar tests. When more than two time points are compared, multiple testing and Bonferroni correction

will be applied.

For metric data, comparisons will be done using Wilcoxon signed-rank tests. If more than two time points are compared Freidman tests will be used. Should significant differences be detected by Friedman, multiple Wilcoxon signed-rank tests will be used as post-hoc tests in combination with Bonferroni correction.

Should baseline data of subgroups be compared (e.g. male/female, patients with or without certain medication) Chi-square or Fischer exact tests for categorical variables and Mann-Whitney-tests or Kruskal-Wallis tests (for two or more groups, respectively) for metric data will be applied.

Descriptive statistics will be given as median (1st quartile, 3rd quartile).

For Microbiome data:

To compare the abundance of OTUs or higher ranked phylogenetic taxa, Mann-Whitney tests will be applied. Benjamini-Hochberg procedure will be used to correct for multiple testing, allowing for a 5% false discovery rate.

Various Machine learning techniques (e.g. Random Forest Classifier, LDA effect size) will be used to identify OTUs that are most descriptive of the microbiome changes by the probiotic intervention. Differentially abundant OTUs/OTUs of interest will be treated like non-microbiome data by extracting their absolute or relative abundance.

For both data types:

Pearson or Spearman correlation (as appropriate) will be used to determine associations between variables. If applicable, cut-offs will be calculated using Youden index.

P-values below 0.05 will be considered significant.

3.3. Gender aspects in research approach

We will aim to offer equal opportunities for all patients willing to participate and we will include gender as a variable in our statistical analysis plan.

3.4. Description of methods to be applied

3.4.1. Sample *acquisition*

All study related procedures will be done after obtaining written informed consent.

Study visits will be on outpatient basis. Routine blood biochemistry analysis including full blood count, electrolytes, renal function, liver function, blood clotting, vitamin B12 levels and C-reactive protein will be performed as part of the routine patient care and data will be recorded for the study.

3.4.2. Questionnaires

Patients will be asked to fill out various questionnaires on quality of life and digestive complaints during all time points:

- Questionnaire on the gastrointestinal quality of life according to Eyparsch
- SF36 (standardized questionnaire on health related quality of life)

3.4.3. Blood sampling

Peripheral venous blood is aseptically collected into pyrogen-free tubes (VACUETTE®, Greiner Bio-One, Kremsmuenster, Austria) and kept at 4°C. For harvesting plasma, blood is centrifuged at 2000xg for 10 min, for serum blood is kept at room temperature for 30 minutes, and then centrifuged. After centrifugation, plasma or serum is aliquoted under pyrogen-free conditions into non-pyrogenic cryotubes (Eppendorf, Hamburg, Germany) and stored at -80°C until further analysis.

3.4.4. Calprotectin:

A ready-to-use solid-phase sandwich ELISA (Immundiagnostik AG, Bensheim, Germany) is used to detect Calprotectin in stool samples. The test is performed according to the manufacturer's instructions. Briefly, thawed stool samples are diluted in extraction buffer by means of special stool extraction system (Immundiagnostik AG, Bensheim, Germany). Supernatant of stool suspension, standards and controls are immobilized at the per-coated plate. After incubated with conjugate, substrate solution is added. The reaction is stopped by addition of stop solution and measurement is done at 450nm versus 690nm as reference wavelength.

3.4.5. Microbiome analysis:

Total DNA from frozen stool samples is isolated with the MagnaPure LC DNA Isolation Kit III (Bacteria, Fungi) (Roche, Mannheim, Germany) according to manufacturer's instructions. Samples are thawed and homogenized in 500µl of

bacterial lysis buffer (Roche, Mannheim, Germany). 250µl of homogenized stool sample are transferred to a Magna Lyser green bead tubes (Roche, Mannheim Germany) and bead beated for mechanical lysis at 6500 rpm for 30 seconds two times in a MagNA Lyser Instrument (Roche, Mannheim Germany). After mechanic lysis enzymatic lysis with 20µl lysozyme at 37°C for 30 minutes and 30µl Proteinase K for 1.5 hours at 65°C is performed. Proteinase K is heat inactivated at 95°C for 10 minutes. The remaining steps are performed according to instructions from the Magna Pure DNA isolation kit and 250µl of the sample were used for DNA purification in a MagnaPure instrument. Total DNA is eluted in 100µl elution buffer and stored at -20°C until PCR amplification. For target specific PCR amplification of hypervariable regions, the primers 27F and R357 are used and synthesized at Eurofins (MWG, Ebersberg, Germany) (27F-AGAGTTTGATCCTGGCTCAG; R357-CTGCTGCCTYCCGTA). 2µl of total DNA are used in a 25µ PCR reaction in triplicates with containing 1 x Fast Start High Fidelity Buffer (Roche, Mannheim, Germany), 1.25 U High Fidelity Enzyme (Roche, Mannheim, Germany), 200 µM dNTPs (Roche, Mannheim, Germany), 0.4 µM barcoded primers and PCR-grade water (Roche, Mannheim, Germany). Thermal Cycling 95°C for 3 minutes, 30 cycles: 95°C for 45 seconds, 55°C for 45 72°C for 1 minute, final extension at 72°C for 7 minutes. Triplicates are pooled, checked on a 1% agraose gel and 15µl of pooled PCR product are normalized according to manufacturer’s instructions on a SequalPrep Normalization Plate (Life Technologies). 15µl of the normalized PCR product are used as template for indexing PCR in a 50µl single reaction (composition as described for the targeted PCR) to introduce barcode sequences to each sample (according to Kozich et al. 2013). Cycling conditions are the same as for the targeted PCR with only 8 cycles for amplification. After indexing 5µl of each sample are pooled, 50µ of the unpurified library are loaded to a 1% agarose gel and purified from the gel with a Qiaquick Gel Extraction Kit (Qiagen, Hilden, Germany) according to manufacturer’s instructions. The pool is quantified using the QuantiFluor ONE dsDNA Dye on the Promega Quantus instrument according to manufacturer’s instructions and size of sequencing library is validation on an Agilent 2100 Bioanalyzer (Agilent) using a high sensitivity DNA assay according to manufacturer’s instructions. The pool containing all samples is run at 6pM final concentration with version 3 600 cycles chemistry

(Illumina, Eindhoven, Netherlands) according to manufacturer‘s and with 20% PhiX control DNA (Illumina, Eindhoven, Netherlands). FASTQ files are used for data analysis.

Data analysis is performed with QIIME1.9.1 tool implemented on a local Galaxy instance (https://galaxy.medunigraz.at/). Paired end reads are joined by fastq-join tool. Primers are removed by cutadapt 1.6 and USEARCH 6.1 is used for reference (SILVA v123) based chimera detection. Open reference (SILVA v123) operational taxonomic unit (OTU) picking is done to define present OTUs. Clustering is performed by UCLUST with a 97% sequence similarity threshold. Fasttree is used to generate a phylogenetic tree. For statistical analysis of OTU abundance OTU table is filtered for all OTUs that reach at least 0.05% of all reads and are present in at least two samples. Chao1 index is used to analyze alpha rarefaction and diversity. Beta diversity analysis is based on Bray Curtis dissimilarity. For OTU abundance comparisons Mann-Whitney-U-tests were performed followed by Benjamini-Hochberg correction to account for multiple testing.

The web-based program Calypso 7.14 (http://cgenome.net/calypso/) was used for RDA and LEfSe calculations as well as the visualization of thereby obtained results.

3.4.6. LBP

A ready-to-use solid-phase sandwich ELISA (Hycult biotechnology, Uden, Netherlands) is used to detect LBP levels in EDTA plasma samples. The test is performed according to the manufacturer’s instructions. Briefly, samples and standards are bound to the pre-coated plate. Afterwards samples and standards are incubated with tracer antibody followed by a streptavidin-peroxidase solution. After incubation with TMB substrate the reaction is stopped by addition of stop solution and measurement is performed at 450nm.

3.4.7. LPS

The presence of endotoxin will be detected via a HEK-blue reporter cell line that is stably transfected with a TLR4 reporter cassette. The activation of TLR4 triggers NFkB expression and subsequently the secretion of secretory embryonic alkaline phosphatase HEK-Blue™ LPS Detection Kit (Invivogen, Toulouse, France) with an adapted protocol is used. (Horvath A, et al. 2016) This assay is based on the ability of

TLR4 to recognize structurally different LPS from gram-negative bacteria. In brief, cells are cultured in 24-well plates (5x104/well). After 24 hours medium is discarded and replaced with samples/LPS standards and detection medium. Cells are incubated for 24 hours at 37°C and colour intensity is measured at a wave length of 650nm.

3.4.8. Zonulin

A ready-to-use solid-phase sandwich ELISA (Immundiagnostik AG, Bensheim, Germany) is used to detect Zonulin in stool samples. The test is performed according to the manufacturer's instructions. Briefly, thawed stool samples are diluted in extraction buffer by means of special stool extraction system (Immundiagnostik AG, Bensheim, Germany). Supernatant of stool suspension, standards and controls are mixed with tracer and then immobilized at the pre-coated plate. After incubated with conjugate, substrate solution is added. The reaction is stopped by addition of stop solution and measurement is done at 450nm versus 690nm as reference wavelength

3.4.9. sCD14

A ready-to-use solid-phase sandwich ELISA (R&D Systems, Abbington, UK) was used to detect sCD14 levels in EDTA plasma samples. The test is performed according to the manufacturer's instructions. Briefly, samples and standards were bound to the pre-coated plate. Afterwards samples and standards were incubated with tracer antibody followed by a streptavidin-peroxidase solution. After incubation with TMB substrate the reaction was stopped by addition of stop solution and measurement was performed at 450nm.

4. Work and Time Plan

Task / Months	1-3	4-6	7-9	10-12
Study initiation	x			
Patient recruitment	x	x		
Patient follow up	x	x	X	
Evaluation of experiments;		x	x	x

data preparation for publication and presentation				
Data presentation on scientific meetings, thesis writing				x

5. General rules and provisions

5.1. Ethical aspects

The study product is considered to be safe patients with long-term PPI use. At best, the participation in this study may even prevent side effects of PPIs. The planned blood sampling and stool collections are minimal stressors. In our opinion, the possible benefit for the patients (positive effect by the probiotic administered) is larger than the inconvenience of the planned procedures.

5.2. Legal regulations and provisions relevant to the study

The study will be performed in accordance to the ICH-GCP guidelines. All documents will be checked and external data monitoring will be performed. All team members directly involved with patient care in this study hold a valid GCP training certificate.

6. Potential additional benefits of the study

The short-term potential of this project will be the generation of first data on the effect of probiotics to revert PPI induced dysbiosis and its consequences, leading to high quality scientific publications and increasing the visibility of clinical research from Austria. In the mid- and long-term the results of this study – if the hypothesis holds true – will lead to the design and conduct of further, larger, intervention studies in this field. The long-term aim will be to produce a large body of evidence to allow general recommendations on novel treatment or prevention strategies taking into account the intestinal microbiome of patients with long-term PPI use.

Literature

[1] “Top 100 Drugs for Q4 2013 by Sales - U.S. Pharmaceutical Statistics.” [Online]. Available: https://www.drugs.com/stats/top100/sales. [Accessed: 27-Jun-2017].

[2] M. Bardou, Y. Toubouti, D. Benhaberou-Brun, E. Rahme, and A. N. Barkun, “Meta-analysis: proton-pump inhibition in high-risk patients with acute peptic ulcer bleeding,” *Aliment. Pharmacol. Ther.*, vol. 21, no. 6, pp. 677–686, Mar. 2005.

[3] V. K. Sharma, G. I. Leontiadis, and C. W. Howden, “Meta-analysis of randomized controlled trials comparing standard clinical doses of omeprazole and lansoprazole in erosive oesophagitis.,” *Aliment. Pharmacol. Ther.*, vol. 15, no. 2, pp. 227–31, Feb. 2001.

[4] K. R. DeVault, D. O. Castell, and American College of Gastroenterology, “Updated Guidelines for the Diagnosis and Treatment of Gastroesophageal Reflux Disease,” *Am. J. Gastroenterol.*, vol. 100, no. 1, pp. 190–200, Jan. 2005.

[5] P. S. Yachimski, E. A. Farrell, D. P. Hunt, and A. E. Reid, “Proton pump inhibitors for prophylaxis of nosocomial upper gastrointestinal tract bleeding: effect of standardized guidelines on prescribing practice.,” *Arch. Intern. Med.*, vol. 170, no. 9, pp. 779–83, May 2010.

[6] G. Singh and G. Triadafilopoulos, “Appropriate choice of proton pump inhibitor therapy in the prevention and management of NSAID-related gastrointestinal damage,” *Int. J. Clin. Pract.*, vol. 59, no. 10, pp. 1210–1217, Sep. 2005.

[7] R. Jung and R. MacLaren, “Proton-Pump Inhibitors for Stress Ulcer Prophylaxis in Critically Ill Patients,” *Ann. Pharmacother.*, vol. 36, no. 12, pp. 1929–1937, Dec. 2002.

[8] E. G. McDonald, J. Milligan, C. Frenette, and T. C. Lee, “Continuous Proton Pump Inhibitor Therapy and the Associated Risk of Recurrent Clostridium difficile Infection.,” *JAMA Intern. Med.*, vol. 175, no. 5, pp. 784–91, May 2015.

[9] J. Leonard, J. K. Marshall, and P. Moayyedi, “Systematic Review of the Risk of Enteric Infection in Patients Taking Acid Suppression,” *Am. J. Gastroenterol.*, vol. 102, no. 9, pp. 2047–2056, Sep. 2007.

[10] S. Janarthanan, I. Ditah, D. G. Adler, and M. N. Ehrinpreis, “Clostridium difficile-Associated Diarrhea and Proton Pump Inhibitor Therapy: A Meta-Analysis,” *Am. J. Gastroenterol.*, vol. 107, no. 7, pp. 1001–1010, Jul. 2012.

[11] D. Compare *et al.*, “Effects of long-term PPI treatment on producing bowel symptoms and SIBO,” *Eur. J. Clin. Invest.*, vol. 41, no. 4, pp. 380–386, Apr. 2011.

[12] S. P. Marcuard, L. Albernaz, and P. G. Khazanie, “Omeprazole therapy causes malabsorption of cyanocobalamin (vitamin B12),” *Ann. Intern. Med.*, vol. 120, no. 3, pp. 211–5, Feb. 1994.

[13] J. R. Saltzman, J. A. Kemp, B. B. Golner, M. C. Pedrosa, G. E. Dallal, and R. M. Russell, “Effect of hypochlorhydria due to omeprazole treatment or atrophic gastritis on protein-bound vitamin B12 absorption.,” *J. Am. Coll. Nutr.*, vol. 13, no. 6, pp. 584–91, Dec. 1994.

[14] R. J. Valuck and J. M. Ruscin, “A case-control study on adverse effects: H2 blocker or proton pump inhibitor use and risk of vitamin B12 deficiency in older adults,” *J. Clin. Epidemiol.*, vol. 57, no. 4, pp. 422–428, Apr. 2004.

[15] Y. Xie, B. Bowe, T. Li, H. Xian, Y. Yan, and Z. Al-Aly, “Risk of death among users of Proton Pump Inhibitors: a longitudinal observational cohort study of United States veterans,” *BMJ Open*, vol. 7, no. 6, p. e015735, Jun. 2017.

[16] C. G. Buffie and E. G. Pamer, “Microbiota-mediated colonization resistance against intestinal pathogens,” *Nat. Rev. Immunol.*, vol. 13, no. 11, pp. 790–

801, Oct. 2013.

[17] N. Kamada, G. Y. Chen, N. Inohara, and G. N??ez, “Control of pathogens and pathobionts by the gut microbiota,” *Nat. Immunol.*, vol. 14, no. 7, pp. 685–690, Jun. 2013.

[18] R. A. Britton and V. B. Young, “Role of the Intestinal Microbiota in Resistance to Colonization by Clostridium difficile,” *Gastroenterology*, vol. 146, no. 6, pp. 1547–1553, May 2014.

[19] C. G. Buffie *et al.*, “Precision microbiome reconstitution restores bile acid mediated resistance to Clostridium difficile.,” *Nature*, vol. 517, no. 7533, pp. 205–8, Jan. 2015.

[20] C. Manichanh *et al.*, “Anal gas evacuation and colonic microbiota in patients with flatulence: effect of diet,” *Gut*, vol. 63, no. 3, pp. 401–408, Mar. 2014.

[21] A. M. Schubert *et al.*, “Microbiome Data Distinguish Patients with Clostridium difficile Infection and Non-C. difficile-Associated Diarrhea from Healthy Controls,” *MBio*, vol. 5, no. 3, pp. e01021-14-e01021-14, May 2014.

[22] B. Hippe *et al.*, “Abundance and diversity of GI microbiota rather than IgG4 levels correlate with abdominal inconvenience and gut permeability in consumers claiming food intolerances.,” *Endocr. Metab. Immune Disord. Drug Targets*, vol. 14, no. 1, pp. 67–75, Mar. 2014.

[23] F. Imhann *et al.*, “Proton pump inhibitors affect the gut microbiome,” *Gut*, vol. 65, no. 5, pp. 740–748, May 2016.

[24] M. A. Jackson *et al.*, “Proton pump inhibitors alter the composition of the gut microbiota,” *Gut*, vol. 65, no. 5, pp. 749–756, May 2016.

[25] M. Lamprecht *et al.*, “Probiotic supplementation affects markers of intestinal barrier, oxidation, and inflammation in trained men; a randomized, double-blinded, placebo-controlled trial,” 2012.

[26] E. Bruzzese *et al.*, “Intestinal inflammation is a frequent feature of cystic fibrosis and is reduced by probiotic administration,” *Aliment. Pharmacol. Ther.*, vol. 20, no. 7, pp. 813–819, Oct. 2004.

[27] A. C. Ouwehand, K. Tiihonen, M. Saarinen, H. Putaala, and N. Rautonen, “Influence of a combination of Lactobacillus acidophilus NCFM and lactitol on healthy elderly: intestinal and immune parameters,” *Br. J. Nutr.*, vol. 101, no. 3, p. 367, Feb. 2009.

[28] N. P. West *et al.*, “Probiotic supplementation for respiratory and gastrointestinal illness symptoms in healthy physically active individuals,” *Clin. Nutr.*, vol. 33, no. 4, pp. 581–587, Aug. 2014.

[29] A. C. Ouwehand *et al.*, “Probiotics reduce symptoms of antibiotic use in a hospital setting: A randomized dose response study,” *Vaccine*, vol. 32, no. 4, pp. 458–463, Jan. 2014.
